# Prognostic significance of the preoperative systemic immune‐inflammation index in patients with oral cavity squamous cell carcinoma treated with curative surgery and adjuvant therapy

**DOI:** 10.1002/cam4.3650

**Published:** 2020-12-16

**Authors:** Sheng‐Ping Hung, Pei‐Rung Chen, Tsung‐Ying Ho, Kai‐Ping Chang, Wen‐Chi Chou, Ching‐Hsin Lee, Yao‐Yu Wu, Po‐Jui Chen, Chia‐Hsin Lin, Yung‐Chih Chou, Kang‐Hsing Fan, Chien‐Yu Lin, Bing‐Shen Huang, Joseph Tung‐Chieh Chang, Chun‐Chieh Wang, Ngan‐Ming Tsang

**Affiliations:** ^1^ Department of Radiation Oncology, Proton and Radiation Therapy Center Linkou Chang Gung Memorial Hospital Taoyuan Taiwan; ^2^ Department of Anesthesiology Mackay Memorial Hospital Taipei Taiwan; ^3^ Department of Nuclear Medicine and Molecular Imaging Center Chang Gung Memorial Hospital and Chang Gung University Taoyuan Taiwan; ^4^ Department of Otolaryngology‐Head Neck Surgery Linkou Chang Gung Memorial Hospital Chang Gung University at Lin‐Kou Taoyuan Taiwan; ^5^ Department of Hematology‐Oncology Chang Gung Memorial Hospital at Linkou and Chang Gung University College of Medicine Taoyuan Taiwan; ^6^ Department of Radiation Oncology New Taipei Municipal TuCheng Hospital Chang Gung Memorial Hospital and Chang Gung University Taoyuan Taiwan; ^7^ Department of Radiation Oncology, Proton and Radiation Therapy Center Kaohsiung Chang Gung Memorial Hospital Kaohsiung Taiwan

**Keywords:** oral cavity squamous cell carcinoma, prognosis, radiotherapy/chemoradiotherapy, risk factors, systemic immune‐inflammation index

## Abstract

**Objectives:**

To investigate the prognostic value of the preoperative systemic immune‐inflammation index (SII) in patients with oral cavity squamous cell carcinoma (OC‐SCC) treated with curative surgery followed by adjuvant radiotherapy (RT) or chemoradiotherapy (CCRT).

**Materials and Methods:**

We retrospectively reviewed the clinical records of patients with OC‐SCC who received surgery and postoperative adjuvant RT/CCRT between January 2005 and December 2012. Blood samples were drawn in the 2 weeks preceding surgery. SII was calculated by multiplying the absolute neutrophil and platelet counts, and then, divided by the absolute lymphocyte count, and its optimal cutoff value was identified using the Youden’s index. The study endpoints included overall survival (OS), local control (LC), regional control (RC), and distant control (DC).

**Results:**

The study sample consisted of 993 patients (58.8% of them treated with CCRT). The optimal cutoff value for SII was 810.6. A total of 347 (34.9%) study participants had high preoperative SII values. After allowance for potential confounders in multivariable analysis, high SII values were independently associated with less favorable DC **(**adjusted hazard ratio [HR] = 1.683, *p* = 0.001) and OS (adjusted HR = 1.466, *p* < 0.001). No independent association between SII and LC/RC was observed.

**Conclusion:**

Increased SII values predict poor DC and OS in patients with OC‐SCC treated with curative resection and adjuvant RT/CCRT. Owing to the higher risk of systemic failure in this patient group, a thorough follow‐up surveillance schedule may be advisable pending independent confirmation of our data.

## Graphical abstract

The SII is defined as platelet × neutrophil/lymphocyte counts. Increased SII values predict poor distant control and overall survival in patients with oral cancer treated with curative resection and adjuvant RT/CCRT. Owing to the higher risk of systemic failure in this patient group, a tight follow‐up surveillance schedule or adjuvant systemic treatment may be advisable pending independent confirmation of our data.

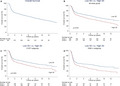

## 1 INTRODUCTION

Growing evidence indicates that inflammation is involved in cancer initiation, progression, and metastasis.^1^ Some readily available parameters originated from routine complete blood count (CBC)––including neutrophils, lymphocytes, monocytes, platelets, and different blood cell ratios––have been found to predict disease recurrence, progression, and survival in patients with head and neck malignancies.^2^, ^3^, ^4^

The systemic immune‐inflammation index (SII)––which is calculated from CBC by multiplying the absolute neutrophil and platelet counts, and then, divided by the absolute lymphocyte count––has prognostic value in several solid tumors.^5^, ^6^, ^7^, ^8^, ^9^, ^10^, ^11^, ^12^ Notably, a previous study^13^ found that high SII values predict less favorable overall survival (OS) and disease‐free survival (DFS) in patients with oral cavity squamous cell carcinoma (OC‐SCC) after curative resection. However, postoperative radiotherapy (RT)––the treatment modality recommended by the National Comprehensive Cancer Network (NCCN) guidelines––was given to less than one‐third of their study patients with nodal metastases.^13^ Concurrent chemoradiotherapy (CCRT) is also recommended for patients with OC‐SCC who carry major pathological risk factors––including positive margins and extranodal extension (ENE).

While both RT and CCRT are known to improve locoregional control, the prognostic value of SII in patients with OC‐SCC who had been treated with curative resection followed by adjuvant RT/CCRT remains unclear. The aim of this retrospective study was twofold, that is, (1) to address this issue using different endpoints related to local, regional, and distant control, and (2) to analyze the predictive ability of SII while taking into account the confounding effect of various clinical and pathological risk factors.

## 2 MATERIALS AND METHODS

### 2.1 Study patients

**FIGURE 1:**
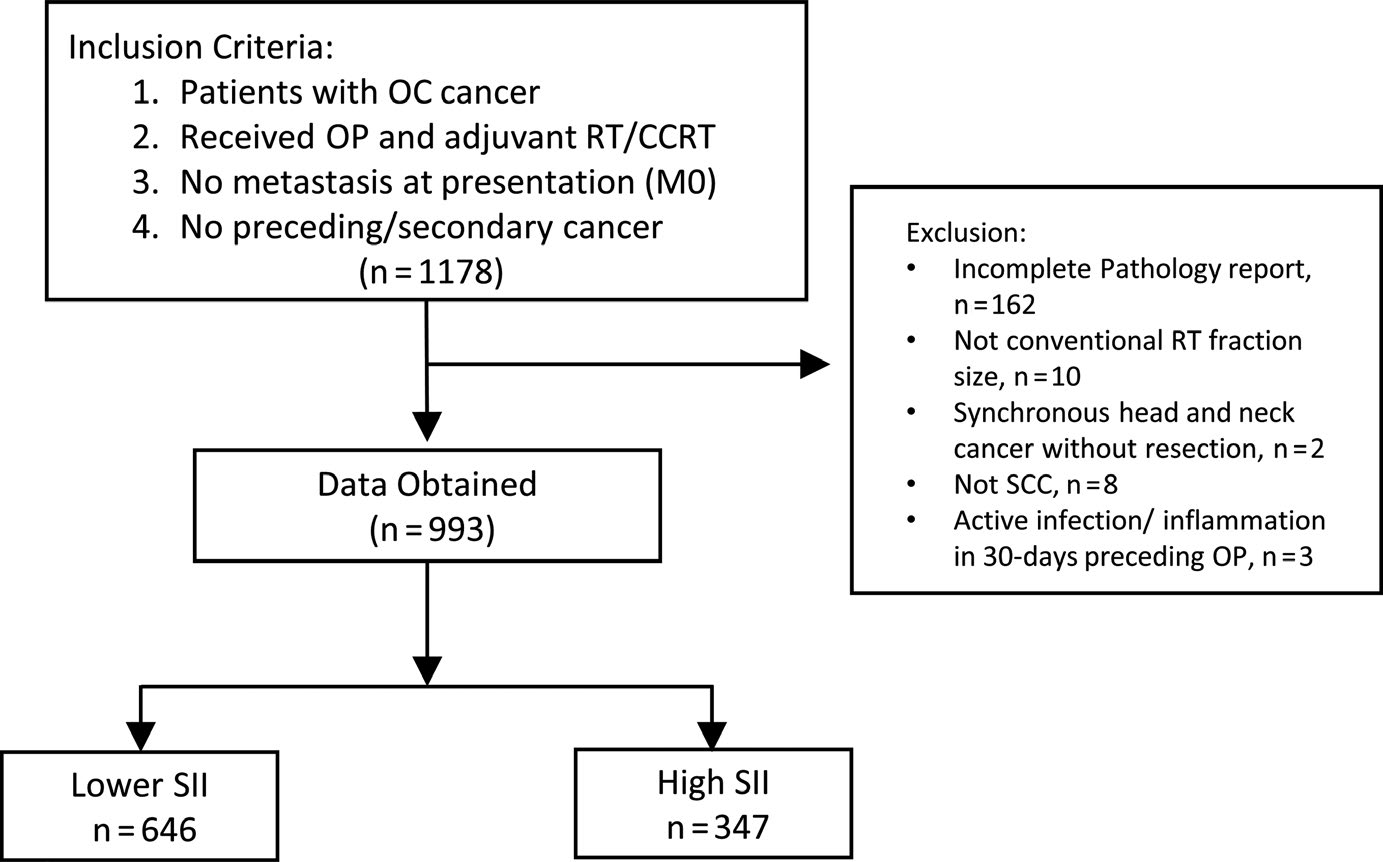
Flow of patients through the study. Abbreviations: OC, oral cavity; OP, operation; RT, radiotherapy; CCRT, chemoradiotherapy; SCC, squamous cell carcinoma

This single‐center study was conducted in a tertiary medical center between 1 January 2005 and 31 December 2012. All of the patients were followed up from the first day of RT until death or censored on the date of last follow‐up (31 July 2019). We identified a total of 1178 patients with nonmetastatic OC‐SCC who met the following inclusion criteria: (1) treatment with radical surgery and adjuvant RT/CCRT and (2) no history of secondary cancer diagnosed in the 3 years preceding or following treatment for OC‐SCC. Exclusion criteria were as follows: (1) incomplete pathology report (margin data were required for inclusion; *n* = 162), (2) unconventional RT dose fraction (*n* = 10), (3) diagnosis of major or minor salivary gland tumors different from SCC, (*n* = 8), (4) synchronous tonsil cancer without curative resection (*n* = 2), and (5) history of an active infectious or inflammatory disorder in the 30 days preceding surgery for OC‐SCC (*n* = 3). Figure 1 depicts the flow of patients through the study. The protocol complied with the tenets of the Helsinki declaration, and ethics approval was granted by the local Institutional Review Board (approval number: 202000259B0). Owing to the retrospective nature of the study, the need for informed consent was waived.

### 2.2 Treatment approach and follow‐up schedule

Patients treated with radical surgery were offered adjuvant therapy (CCRT or RT) according to the presence of specific pathological risk factors. In line with published phase III study,^14^ CCRT was administered to patients with positive surgical margins and/or extranodal extension. Besides, patients having pN2 stage^15^ or presence of at least three minor risk factors^16^ also received CCRT. Minor risk factors included the following variables: pT4 or pN1 stage, close surgical margins (≤ 4 mm), poor differentiation (PD), perineural invasion (PNI), lymphatic invasion, vascular invasion, and tumor depth of invasion (DOI) >10 mm. Patients harboring two minor risk factors or pN1 at level IV−V were given postoperative RT alone. The radiation dose per fraction was 2 Gray (Gy)––with the total dose being 60−66 Gy for adjuvant postoperative RT and 66 Gy for CCRT.^15^ Dose escalation up to 70−72 Gy was allowed for patients who had evidence of early locoregional recurrence confirmed on biopsy or PET/CT imaging before adjuvant treatment. Cisplatin‐based chemotherapy was used in the context of CCRT. All treatment decisions were taken by consensus within a multidisciplinary tumor board consisting of otorhinolaryngologists, radiation oncologists, medical oncologists, radiologists, and pathologists. Posttreatment follow‐up in the first 3 years consisted of medical visits performed every 3 months accompanied by imaging investigations (CT or MRI) every 3−6 months. Thereafter, visits were scheduled every 6 months and imaging studies on a yearly basis.

### 2.3 Calculation of SII and pathological risk factors

A preoperative CBC was retrospectively obtained from blood samples drawn in the 2 weeks preceding surgery for operative evaluation (median interval between blood sampling and operation: 5 days). SII was calculated by multiplying the absolute neutrophil and platelet counts, and then, divided by the absolute lymphocyte count. The following pathological risk factors were collected: surgical margins, extranodal extension (ENE), tumor depth of invasion, perineural invasion, lymphatic invasion, and vascular invasion. Patients were staged using the AJCC Staging Manual, Eight Edition.

### 2.4 Data analysis

Categorical variables were analyzed by the chi‐square test or the Fisher's exact test, as appropriate. Continuous data were compared using the independent Student's *t*‐test (normally distributed variables) or the Mann–Whitney *U* test (skewed data). The frequency of missing data for pathological factors was <5% for all variables, the only exception being perineural invasion (7.6%). Because of the low missing data rate, multiple imputations were not required.^17^ The study endpoints––which included OS, local control (LC), regional control (RC), and distant control (DC)––were calculated from the first day of adjuvant RT/CCRT to the date of the event of interest (or censored on the date of last follow‐up). Local control was defined as the absence of disease recurrence within the original tumor bed––to which a 2‐cm margin was added. Regional control was diagnosed in the absence of regional lymph node (LN) recurrences. Distant control was defined by the absence of disease spread to distant LN or organs. Disease recurrence was diagnosed when recurrent lesions were identified on biopsy or using at least two different imaging modalities. Survival curves were plotted using the Kaplan–Meier method (log‐rank test). Multivariable Cox proportional hazard regression analysis was used to identify independent risk factors for the outcomes of interest. The proportional hazards assumption was tested using graphical diagnostics based on the scaled Schoenfeld residuals and confirmed to be valid. All of the variables associated with the outcomes of interest at a level of *p* < 0.2 in univariate analysis were entered into the multivariable model. The bootstrap method was used for internal validation. Results of multivariable analysis are expressed as adjusted hazard ratios (HRs) with their 95% confidence intervals (CIs). The optimal cutoff value for SII (810.6 in our study) was calculated with the Youden's index. All calculations were performed using the SPSS software package, version 20 (IBM). Two‐tailed *p* values <0.05 were considered statistically significant. The presentation of this article follows the REMARK guidelines.^18^

## 3 RESULTS

### 3.1 Patient characteristics and treatment outcomes

**FIGURE 2:**
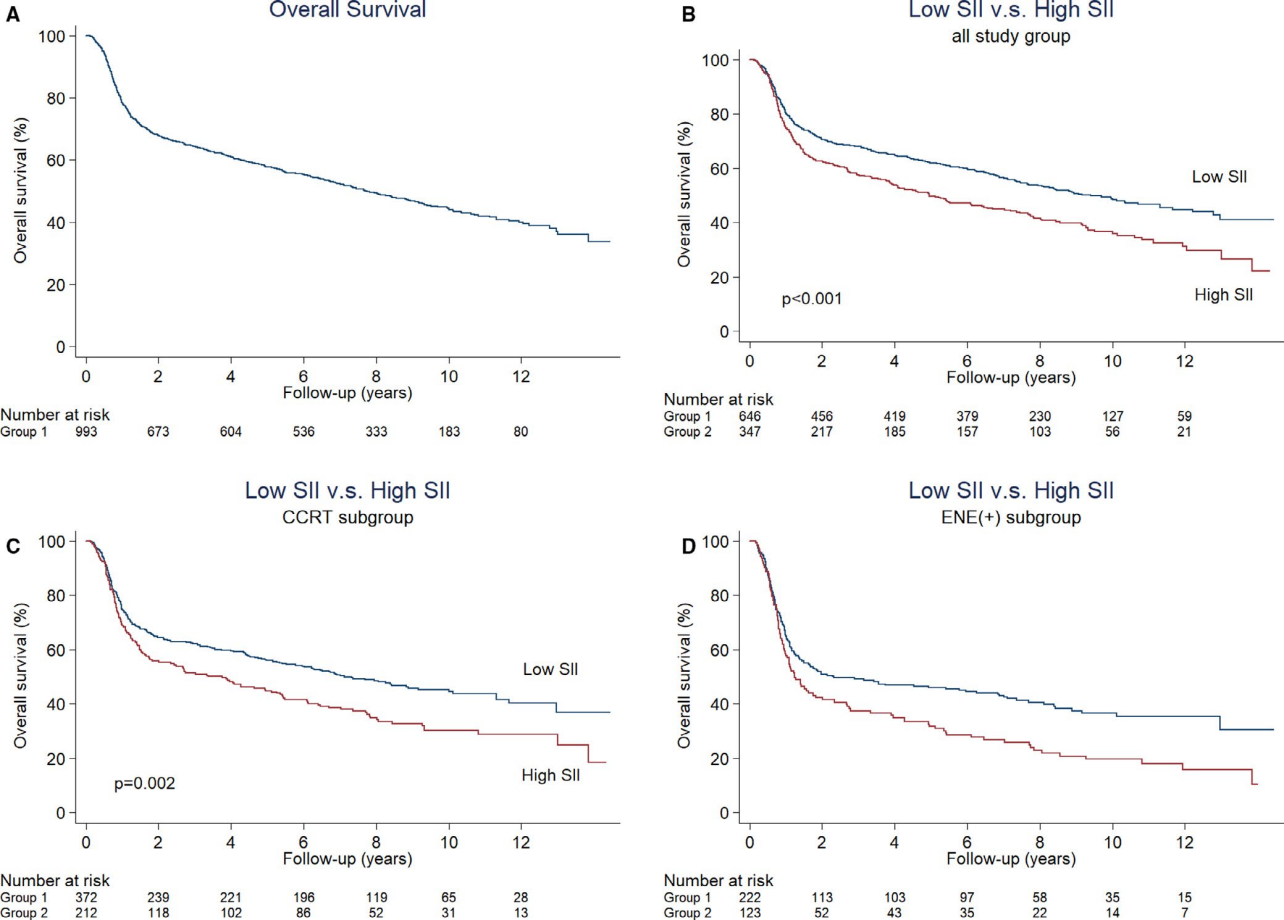
(A) overall survival in the entire study cohort (median: 7.7 years); (B) overall survival of patients with high versus low SII values (median: 4.9 years vs. 9.4 years, respectively, *p* < 0.001); (C) overall survival of patients with high versus low SII values in the subgroup treated with chemoradiotherapy (median: 3.4 vs. 7.1 years, respectively, *p* = 0.002); (D) overall survival of patients with high versus low SII values in the subgroup with extranodal extension (median: 1.2 vs. 2.3 years, respectively, *p* = 0.003)

The study sample consisted of 993 patients (92.8% men, median age: 51 years) with pathologically diagnosed OC‐SCC (Table 1). The three most common tumor sites were as follows: tongue (37.3%), buccal mucosa (34.1%), and gum (15.6%). Advanced T‐ or N‐ stages were identified in 87.8% and 46.8% of the study patients, respectively. Vascular invasion, lymphatic invasion, ENE, perineural invasion, and positive margins were identified in 4.9%, 6.4%, 34.7%, 51.3%, and 4.3% of patients, respectively. Adjuvant RT was given to all participants, and 58.8% of them received CCRT. The median dose used for RT was 66 Gy. The median duration of follow‐up for patients who survived was 8.8 years. The 5‐year and 10‐year OS rates in the entire study cohort were 57.6% and 44.4%, respectively––with a median OS of 7.7 years (Figure 2A). The 5‐year and 10‐year LC, RC, and DC rates were 78.0%/75.5%, 85.1%/84.8%, and 80.6%/80.0%, respectively. The results of univariate and multivariable analyses for the outcomes of interest are summarized in Tables 2 and 3.

**TABLE 1:** General characteristics of the study participants

	Entire cohort (*N* = 993)	Low SII (*N* = 646)	High SII (*N* = 347)	*p*
Sex				
Men	922 (92.8%)	597 (92.4%)	325 (93.7%)	0.468
Women	71 (7.2%)	49 (7.6%)	22 (6.3%)	
Age				
<65 years	893 (89.9%)	577 (89.3%)	316 (91.1%)	0.383
≥65 years	100 (10.1%)	69 (10.7%)	31 (8.9%)	
**pT stage**				
**Stage 1−2**	**121 (12.2%)**	**102 (15.8%)**	**19 (5.5%)**	<**0.001**
**Stage 3−4**	**872 (87.8%)**	**544 (84.2%)**	**328 (94.5%)**	
pN stage				
Stage 0−1	528 (53.2%)	344 (53.3%)	184 (53.0%)	0.946
Stage 2−3	465 (46.8%)	302 (46.7%)	163 (47.0%)	
**p Stage**				
**Stage 1−2**	**35 (3.5%)**	**30 (4.6%)**	**5 (1.4%)**	**0.009**
**Stage 3−4**	**958 (96.5%)**	**616 (95.4%)**	**342 (98.6%)**	
Alcohol drinking	636 (64.0%)	408 (63.2%)	228 (65.7%)	0.425
Betel quid chewing	775 (78.0%)	494 (76.5%)	281 (81.0%)	0.102
Cigarette smoking	862 (86.8%)	528 (86.4%)	304 (87.6%)	0.585
**Tumor site**				
**Tongue**	**370 (37.3%)**	**254 (39.3%)**	**116 (33.4%)**	**0.041**
**Buccal mucosa**	**339 (34.1%)**	**200 (31.0%)**	**139 (40.1%)**	
**Gum**	**155 (15.6%)**	**100 (15.5%)**	**55 (15.9%)**	
**Retromolar**	**53 (5.3%)**	**37 (5.7%)**	**16 (4.6%)**	
**Mouth floor**	**37 (3.7%)**	**27 (4.2%)**	**10 (2.9%)**	
**Lip**	**20 (2.0%)**	**17 (2.6%)**	**3 (0.9%)**	
**Hard palate**	**19 (1.9%)**	**11 (1.7%)**	**8 (2.3%)**	
**WHO differentiation**				<**0.001**
**Well differentiated**	**243 (24.5%)**	**142 (22.0%)**	**101 (29.1%)**	
**Moderately differentiated**	**613 (61.7%)**	**427 (66.2%)**	**186 (53.6%)**	
**Poorly differentiated**	**136 (13.7%)**	**76 (11.7%)**	**60 (17.3%)**	
N/A	1 (0.1%)	1 (0.1%)	—	
Vascular invasion	49 (4.9%)	33 (5.1%)	16 (4.6%)	0.679
N/A	27 (2.7%)	22 (3.4%)	5 (1.4%)	
Lymphatic invasion	64 (6.4%)	39 (6.0%)	25 (7.2%)	0.527
N/A	30 (3.0%)	24 (3.7%)	6 (1.7%)	
Extranodal extension	345 (34.7%)	222 (34.4%)	123 (35.4%)	0.733
Margin positives	43 (4.3%)	27 (4.2%)	16 (4.6%)	0.750
Perineural invasion	509 (51.3%)	322 (49.8%)	187 (53.9%)	0.744
N/A	75 (7.6%)	61 (9.4%)	14 (4.0%)	
**Depth of invasion **>**10 mm**	**723 (72.8%)**	**428 (66.8%)**	**295 (85.3%)**	<**0.001**
N/A	6 (0.6%)	5 (0.8%)	1 (0.3%)	
CCRT	584 (58.8%)	372 (57.6%)	212 (61.1%)	0.284
**Early recurrence before RT**	**17 (1.7%)**	**4 (0.3%)**	**13 (3.7%)**	<**0.001**
Surgery−RT interval >6 weeks	299 (30.1%)	191 (29.6%)	108 (31.1%)	0.610
Total RT time >8 weeks	123 (12.4%)	77 (11.9%)	46 (13.3%)	0.542
Median RT dose (EQD2), Gy	66	66	66	0.079

Data are given as counts (percentages in parentheses), unless otherwise indicated. *p* values for differences between the high and low SII groups were calculated with the chi‐square test. Statistically significant variables are marked in bold. Abbreviations: CCRT, concurrent chemoradiotherapy; N/A, not available, missing data; PET, positron emission tomography; RT, radiotherapy; SII, systemic immune‐inflammation index; WHO, World Health Organization.

**TABLE 2:** Univariate analysis of overall survival, local control, regional control, and distant control

	OS (HR, 95% CI) *p*		LC (HR, 95% CI) *p*		RC (HR, 95% CI) *p*		DC (HR, 95% CI)	
Age (≥65 vs. <65 years)	1.263 (0.973–1.639)	0.080	0.622 (0.355–1.092)	0.098	0.884 (0.489–1.599)	0.684	0.767 (0.444–1.323)	0.340
Sex (men vs. women)	0.951 (0.689–1.312)	0.759	0.839 (0.511–1.380)	0.489	0.881 (0.475–1.631)	0.686	1.018 (0.579–1.791)	0.949
pT stage (1−2 vs. 3−4)	**1.483 (1.114–1.974)**	**0.007**	1.496 (0.932–2.400)	0.095	1.406 (0.794–2.491)	0.242	1.545 (0.925–2.582)	0.097
pN stage (0−1 vs. 2−3)	**1.858 (1.567–2.203)**	<**0.001**	**1.467 (1.111–1.937)**	**0.007**	**2.471 (1.737–3.515)**	<**0.001**	**3.525 (2.547–4.877)**	<**0.001**
CCRT	**1.505 (1.262–1.795)**	<**0.001**	**1.416 (1.061–1.888)**	**0.018**	**1.724 (1.198–2.481)**	**0.003**	**2.113 (1.521–2.934)**	<**0.001**
Alcohol drinking	**1.375 (1.143–1.654)**	**0.001**	**1.432 (1.056–1.940)**	**0.021**	**1.710 (1.164–2.511)**	**0.006**	1.299 (0.947–1.780)	0.105
Betel quid chewing	1.117 (0.907–1.376)	0.296	**1.513 (1.041–2.197)**	**0.030**	0.995 (0.664–1.493)	0.983	1.291 (0.885–1.883)	0.185
Cigarette smoking	0.945 (0.738–1.209)	0.651	1.056 (0.695–1.606)	0.798	0.983 (0.598–1.613)	0.945	1.360 (0.836–2.213)	0.216
Location (vs. tongue)								
Buccal mucosa	1.085 (0.885–1.330)	0.431	1.181 (0.849–1.643)	0.324	0.816 (0.559–1.192)	0.293	1.404 (0.995–1.982)	0.054
Gum	1.123 (0.875–1.441)	0.363	1.178 (0.781–1.778)	0.435	0.606 (0.350–1.049)	0.074	0.967 (0.601–1.557)	0.892
Retromolar	1.020 (0.694–1.500)	0.920	1.152 (0.624–2.128)	0.652	0.512 (0.206–1.275)	0.150	1.245 (0.653–2.372)	0.505
Mouth floor	0.958 (0.598–1.536)	0.859	0.267 (0.066–1.091)	0.066	0.148 (0.021–1.067)	0.058	0.990 (0.427–2.294)	0.981
Lip	1.314 (0.749–2.305)	0.342	1.311 (0.529–3.251)	0.559	0.866 (0.272–2.760)	0.808	1.253 (0.455–3.450)	0.663
Hard palate	1.550 (0.900–2.671)	0.114	1.978 (0.858–4.558)	0.109	0.633 (0.155–2.589)	0.525	1.406 (0.510–3.872)	0.510
Differentiation MD versus WD	**1.271 (1.033–1.565)**	**0.023**	1.159 (0.834–1.610)	0.379	**1.730 (1.096–2.731)**	**0.019**	**1.793 (1.187–2.708)**	**0.006**
PD versus WD	1.241 (0.925–1.665)	0.149	0.822 (0.489–1.383)	0.460	1.727 (0.949–3.145)	0.074	**2.455 (1.489–4.050)**	<**0.001**
Surgery−RT interval >6 weeks	**1.336 (1.120–1.593)**	**0.001**	**1.564 (1.176–2.080)**	**0.002**	1.378 (0.971–1.955)	0.073	1.088 (0.793–1.492)	0.601
Total RT time >8 weeks	**1.876 (1.495–2.353)**	<**0.001**	1.343 (0.889–2.030)	0.161	1.548 (0.980–2.445)	0.061	**1.585 (1.064–2.359)**	**0.023**
Positive margin	**1.959 (1.369–2.803)**	<**0.001**	**2.511 (1.505–4.188)**	<**0.001**	1.731 (0.880–3.405)	0.112	1.175 (0.578–2.388)	0.656
Extranodal extension	**2.068 (1.743–2.452)**	<**0.001**	**1.502 (1.127–2.003)**	**0.006**	**2.441 (1.742–3.421)**	<**0.001**	**3.968 (2.933–5.369)**	<**0.001**
Early Recurrence before RT	**2.466 (1.421–4.281)**	**0.001**	2.186 (0.899–5.315)	0.085	1.623 (0.517–5.099)	0.407	2.276 (0.935–5.542)	0.070
Perineural invasion	**1.199 (1.003–1.432)**	**0.046**	1.028 (0.767–1.378)	0.853	1.313 (0.919–1.877)	0.134	**1.807 (1.308–2.497)**	<**0.001**
Vascular invasion	1.256 (0.864–1.827)	0.233	0.788 (0.370–1.676)	0.536	1.663 (0.873–3.168)	0.122	1.693 (0.962–2.979)	0.068
Lymphatic invasion	**1.951 (1.437–2.650)**	<**0.001**	0.798 (0.393–1.619)	0.531	1.395 (0.732–2.659)	0.311	**2.522 (1.598–3.980)**	<**0.001**
Depth of invasion >10 mm	**1.431 (1.170–1.750)**	<**0.001**	1.055 (0.773–1.439)	0.735	1.171 (0.794–1.727)	0.425	**1.584 (1.099–2.282)**	**0.014**
High SII	**1.417 (1.193–1.683)**	<**0.001**	1.270 (0.954–1.690)	0.102	0.984 (0.690–1.404)	0.930	**1.586 (1.182–2.128)**	**0.002**

Statistically significant variables are marked in bold. Abbreviations: CCRT, chemoradiotherapy; CI, confidence interval; DC, distant control; HR, hazard ratio; LC, local control; MD, moderately differentiated; OS, overall survival; PD, poorly differentiated; RC, regional control; RT, radiotherapy; SII, systemic immune‐inflammation index; WD, well differentiated.

**TABLE 3:** Multivariable analysis of overall survival, local control, regional control, and distant control

	Adjusted HR (95% CI)	*p*	Bootstrap *p*
Overall survival			
Alcohol drinking	1.308 (1.072–1.595)	0.008	0.011
Total RT time >8 weeks	1.616 (1.265–2.065)	<0.001	0.001
Positive margin	2.055 (1.416–2.981)	<0.001	0.002
Extranodal extension	1.897 (1.582–2.274)	<0.001	0.001
Lymphatic invasion	1.747 (1.276–2.392)	0.001	0.007
High SII	1.466 (1.225–1.755)	<0.001	0.002
Local control			
Alcohol drinking	1.394 (1.025–1.895)	0.034	0.027
Surgery−RT interval >6 weeks	1.473 (1.104–1.964)	0.008	0.011
Extranodal extension	1.468 (1.100–1.959)	0.009	0.004
Positive margin	2.426 (1.453–4.052)	0.001	0.008
Regional control*			
Alcohol drinking	1.639 (1.105–2.432)	0.014	0.010
Extranodal extension	2.508 (1.764–3.565)	<0.001	0.001
Distant control			
Extranodal extension	3.416 (2.486–4.694)	<0.001	0.001
Perineural invasion	1.494 (1.074–2.077)	0.017	0.019
Lymphatic invasion	1.911 (1.198–3.047)	0.007	0.007
High SII	1.683 (1.239–2.285)	0.001	0.003

Abbreviations: CI, confidence interval; HR, hazard ratio; RT, radiotherapy; SII, systemic immune‐inflammation index.

* pN stage was not entered as a covariate into the multivariable model because of its high collinearity with extranodal extension (Pearson's correlation =0.765, *p* < 0.001).

### 3.2 Risk factors for poor local and regional control

The following unfavorable risk factors for LC were identified in univariate analysis: advanced N stage, alcohol drinking, betel quid chewing, treatment with CCRT, ENE, positive margins, and an interval between surgery and RT >6 weeks. After allowance for potential confounders in multivariable analysis, alcohol drinking (adjusted HR = 1.394, *p* = 0.034; bootstrap validation, *p* = 0.027), an interval between surgery and RT >6 weeks (adjusted HR = 1.473, *p* = 0.008; bootstrap validation, *p* = 0.011), positive margins (adjusted HR = 2.426, *p* = 0.001; bootstrap validation, *p* = 0.008), and ENE (adjusted HR = 1.468, *p* = 0.010; bootstrap validation, *p* = 0.004) were retained in the model as independent risk factors for LC. The following unfavorable risk factors for RC were identified in univariate analysis: advanced N stage, alcohol drinking, ENE, and treatment with CCRT. After allowance for potential confounders in multivariable analysis, alcohol drinking (adjusted HR = 1.639, *p* = 0.014; bootstrap validation, *p* = 0.010) and ENE (adjusted HR = 2.508, *p* < 0.001; bootstrap validation, *p* = 0.001) were retained in the model as independent risk factors for RC.

### 3.3 Distant control and predictive value of SII

Compared with patients with a low SII, those with high SII values had a higher disease burden (i.e., more advanced T stage as well as a higher frequency of poor differentiation, tumor depth of invasion >10 mm, neoplasms of the buccal mucosa, and more early recurrence before adjuvant treatment; Table 1). High SII values were associated with poor DC. Compared with patients with low SII, those with high SII values had a higher 5‐year DC rate (75.4% vs. 83.3%, respectively, *p* = 0.002). Moreover, the median OS of patients with low SII was markedly higher than that of patients with high SII (9.4 years vs. 4.9 years, respectively, *p* < 0.001; Figure 2B). We then conducted a subgroup analysis in high‐risk patients who received CCRT (median OS: 5.5 years). The results revealed that high SII scores were associated with less favorable OS figures even in this subgroup (3.4 years vs. 7.1 years, respectively, *p* = 0.002; Figure 2C). Similar findings were observed in patients with ENE––who are known to have a dismal prognosis (OS in patients with high vs. low SII scores: 1.2 years vs. 2.3 years, respectively, *p* = 0.003; Figure 2D). In multivariable analysis, higher SII values were independently associated with less favorable DC (adjusted HR = 1.683, *p* = 0.001; bootstrap validation, *p* = 0.003) and OS (adjusted HR = 1.466, *p* < 0.001; bootstrap validation, *p* = 0.002). Other significant predictors of OS identified in univariate analysis included advanced T/N stage, treatment with CCRT, alcohol drinking, an interval between surgery and RT >6 weeks, total treatment time >8 weeks, ENE, positive surgical margins, early recurrence before RT, perineural invasion, lymphatic invasion, and depth of tumor invasion >10 mm. Besides SII, the following variables were retained in the multivariable model as independent risk factors for OS: alcohol drinking (adjusted HR = 1.308, *p* = 0.008; bootstrap validation, *p* = 0.011), total treatment time >8 weeks (adjusted HR = 1.616, *p* < 0.001; bootstrap validation, *p* = 0.001), ENE (adjusted HR = 1.897, *p* < 0.001; bootstrap validation, *p* = 0.001), positive surgical margins (adjusted HR = 2.055, *p* < 0.001; bootstrap validation, *p* = 0.002), and lymphatic invasion (adjusted HR = 1.747, *p* = 0.001; bootstrap validation, *p* = 0.007).

## 4 DISCUSSION

Owing to its capacity to improve LC, RC, and OS,^19^ postoperative RT is currently considered as the standard of care for patients with advanced OC‐SCC. Moreover, an analysis of two randomized control trials demonstrated that CCRT improves LC, DFS, and OS in patients with positive surgical margins and ENE.^20^ Unfortunately, the clinical outcomes of patients with OC‐SCC have remained stagnant over the last decade.^21^ Locoregional failure in areas receiving high radiation doses has been described as the most common pattern of disease recurrence following surgery and postoperative RT.^22^ However, the RTOG‐7303 study indicated that distant metastases and secondary primary tumors are the predominant failure patterns after 2 years of follow‐up in patients who underwent postoperative RT.^23^ Because of the clear prognostic relevance of distant failure, tools that may improve its risk stratification are eagerly awaited.

Inflammation and tumorigenesis are closely intertwined and several inflammatory markers may have prognostic significance in patients with malignancies.^1^, ^24^ In this regard, the SII has been extensively investigated in patients with solid tumors––including esophageal cancer,^25^ hepatocellular carcinoma,^12^ urothelial carcinoma of bladder^10^ or upper urinary tract,^26^ cervical cancer,^7^ pancreatic cancer,^5^ and non‐small cell lung cancer.^27^ Published studies consistently reported that SII independently predict OS, but data on its potential association with LC or distant metastases have been scanty. Moreover, the available literature is chiefly focused on patients treated with surgery, the only exception being a study conducted in non‐small cell lung cancer^27^––which also showed that high SII may predict a poor response to RT. A previous study^13^ found that SII is an independent prognostic factor for OS and disease‐free survival in patients with oral cavity cancer. However, the authors did not focus on patients who underwent combination treatment and did not take other known risk factors into account.

The current study––in which all patients with OC‐SCC received adjuvant RT or CCRT after surgery––demonstrated for the first time that high preoperative SII values are associated with a less favorable OS independent of potential confounders. Our data indicate that ENE was the strongest risk factors for adverse clinical outcomes. Notably, OS of patients with and without ENE was markedly different (1.75 years vs. 10.35 years, respectively). Nonetheless, patients with ENE and low SII had a more favorable OS compared with those with high SII values––suggesting that this index may be useful for risk stratification even in this high‐risk subgroup. Similar findings were observed in the subset of patients who underwent CCRT. It is possible that such differences in terms of OS could be driven by a lower 5‐year DC rate in patients with high SII values (75.4% vs. 83.3%). However, SII was not found to be associated with LC and RC––a finding which is in apparent contrast with the previously reported association with high SII and radiation resistance.^27^ Nonetheless, comparisons should be interpreted cautiously because we used RT in an adjuvant setting, whereas it was given as primary treatment in the study by Tong et al.^27^ The goal of adjuvant radiotherapy is to clear microscopic disease foci––which elicit a less prominent inflammatory response compared to gross macroscopic tumors. This observation may offer an explanation for the lack of association between SII and LC/RC in our study. However, we found that SII was independently related with DC. Some mechanisms through which SII may be related to the occurrence of distant metastases are as follows: (1) neutrophil production of tumor necrosis factor (TNF)‐α––a cytokine that impairs CD8+ T cells activity and induces vascular leaking,^24^ (2) platelet production of growth factors that protect malignant cells against natural killer cell‐induced cell death,^28^ and (3) blunted lymphocyte‐mediated immune response against malignant cells.^29^ Of interest, high SII values were related to depth of tumor invasion, advanced T stage, poor differentiation, anatomical location in the buccal mucosa, and early recurrence before RT. While some of these risk factors were associated to less favorable OS and DC figures, SII was the only factor retained in the multivariate model as an independent risk factor. Our findings may pave the way to further investigations on the clinical utility of preoperative SII to identify patients who could benefit from more aggressive treatment strategies. For example, the RTOG‐0234 study^30^ demonstrated that concurrent treatment with docetaxel and cetuximab may reduce the risk of distant metastases compared with the scheme described in the RTOG‐9501 (i.e., the standard treatment currently given to high‐risk patients in our center). Metronomic adjuvant chemotherapy may also reduce distant recurrences.^31^ The utility of these approaches in patients with high preoperative SII values deserves further scrutiny.

Our findings need to be interpreted in the context of some limitations. Owing to the retrospective nature of the study, our investigation is prone to bias and needs independent confirmation in prospective cohorts. While our findings were internally validated using the bootstrap method, we did not conduct an external validation. Thus, the question as to whether our results are generalizable to different setting remains answered. We were unable to investigate the potential association between SII and treatment complications because of missing data related to adverse effects. Moreover, SII was determined on pretreatment blood samples without resorting to serial measurements. Further research is required to investigate whether SII may change during the course of RT or CCRT. We nonetheless believe that the value of pretreatment SII to identify high‐risk patients with oral cavity cancer may be clinically relevant. Previous studies have shown that prolonged treatment duration can have an adverse impact on disease control.^32^, ^33^ In this scenario, an appropriate treatment planning is critical to optimize outcomes. Subject to future validation in prospective studies, SII can be useful to guide treatment decisions. Finally, the optimal cutoff value for SII may vary across different populations.

In conclusion, the results of our study indicate that increased SII values are associated with poor clinical outcomes (OS and DC) in patients with OC‐SCC treated with curative resection and adjuvant RT/CCRT. Owing to the higher risk of systemic failure in this patient group, a thorough follow‐up surveillance schedule may be advisable; future research is required to examine this hypothesis more rigorously.

## CONFLICT OF INTEREST

None.

## FUNDING INFORMATION

This research did not receive any specific grant from funding agencies in the public, commercial, or not‐for‐profit sectors.

## Data Availability

The data sets generated during and/or analyzed during the current study are available from the corresponding author upon reasonable request. Data cannot be made publicly available because of privacy regulations or ethical restrictions.
